# Non-Coding RNAs in HIV Infection, NeuroHIV, and Related Comorbidities

**DOI:** 10.3390/cells13110898

**Published:** 2024-05-23

**Authors:** Seema Singh, Uma Maheswari Deshetty, Sudipta Ray, Abiola Oladapo, Elias Horanieh, Shilpa Buch, Palsamy Periyasamy

**Affiliations:** Department of Pharmacology and Experimental Neuroscience, University of Nebraska Medical Center, Omaha, NE 68198-5880, USA; seema.singh@unmc.edu (S.S.); udeshetty@unmc.edu (U.M.D.); suray@unmc.edu (S.R.); abiola.oladapo@unmc.edu (A.O.); ehoranieh@unmc.edu (E.H.)

**Keywords:** HIV, ncRNAs, miRNAs, lncRNAs, neuroinflammation, neuroHIV

## Abstract

NeuroHIV affects approximately 30–60% of people living with HIV-1 (PLWH) and is characterized by varying degrees of cognitive impairments, presenting a multifaceted challenge, the underlying cause of which is chronic, low-level neuroinflammation. Such smoldering neuroinflammation is likely an outcome of lifelong reliance on antiretrovirals coupled with residual virus replication in the brains of PLWH. Despite advancements in antiretroviral therapeutics, our understanding of the molecular mechanism(s) driving inflammatory processes in the brain remains limited. Recent times have seen the emergence of non-coding RNAs (ncRNAs) as critical regulators of gene expression, underlying the neuroinflammatory processes in HIV infection, NeuroHIV, and their associated comorbidities. This review explores the role of various classes of ncRNAs and their regulatory functions implicated in HIV infection, neuropathogenesis, and related conditions. The dysregulated expression of ncRNAs is known to exacerbate the neuroinflammatory responses, thus contributing to neurocognitive impairments in PLWH. This review also discusses the diagnostic and therapeutic potential of ncRNAs in HIV infection and its comorbidities, suggesting their utility as non-invasive biomarkers and targets for modulating neuroinflammatory pathways. Understanding these regulatory roles could pave the way for novel diagnostic strategies and therapeutic interventions in the context of HIV and its comorbidities.

## 1. Introduction

Human immunodeficiency virus (HIV)-1 is an etiological agent of Acquired Immunodeficiency Syndrome (AIDS), a devastating infectious disease that targets the immune system, primarily infecting the CD4+ T cells and myeloid cells. HIV infection is known to cause a significant decline in CD4+ T cells in the host, resulting in a weakened immune system, in turn increasing the susceptibility of infected individuals to various opportunistic infections and an increased risk of developing certain cancers, collectively referred to as AIDS [1]. Identified several decades ago, the virus has claimed the lives of millions, with approximately 40 million people still living with the infection across the globe [2]. The discovery of combined antiretroviral therapy (cART) has been a boon for those infected with HIV-1. It dramatically reduces viremia, while also increasing the lifespan of those infected with the virus, thereby transforming this devastating disease from a death sentence into a more manageable and chronic condition. Paradoxically, however, an increased lifespan in people living with HIV-1 (PLWH) is also accompanied by disturbances in cognition, premature aging, and, in rare cases, dementia [3]. Despite the effectiveness of cART, viral reservoirs continue to persist in various cells, including CD4+ T cells, hematopoietic stem cells, dendritic cells, and myeloid cells such as microglia [4]. Of these, microglial cells that reside in the brain face unique challenges in combating HIV-1 infection, owing to the anatomical and immunological isolation of the brain via the blood–brain barrier (BBB). Persistent low-level HIV infection in the brain, coupled with cART and possibly drug abuse, have been implicated in trigging local neuroinflammation, inevitably contributing to HIV-associated neurocognitive disorders (HAND) in a substantial percentage of PLWH [5].

In the literature, there are many hypotheses about how HIV enters and infects the brain. One theory proposes the Trojan horse mechanism [5,6], wherein the virus-infected cells (peripheral monocytes and T cells) cross the BBB within the first two weeks of infection and release viral particles, once in the brain. These particles, in turn, can infect the resident microglial cells [5] and, to some extent, the astrocytes [7,8], thus initiating an inflammatory cascade. Another hypothesis suggests that inflammatory cytokines, including tumor necrosis α (TNF-α), promote a paracellular route for HIV to cross the BBB [9]. One of the early HIV proteins, the transactivator of transcription (Tat), has also been found to destabilize the BBB, increasing its permeability and allowing increased numbers of peripheral inflammatory cells to cross the BBB [10,11,12]. Microglia are the known reservoirs for HIV, since some cART regimens have limited access to the brain, with concentrations in the brain being up to 100-fold lower than those in the serum [13].

In the era of cART, which has significantly improved the management of HIV infection by reducing the overall virus replication and improving the overall health outcomes, challenges still persist within the realm of HAND neuropathogenesis, wherein neuroinflammation and synaptic injury stand out as crucial signature features of the syndrome [5]. Persistent neuroinflammation and neuronal toxicity in the brain can be attributed to many factors. These include chronic immune activation, dysregulated cytokine production, oxidative stress, glutamate excitotoxicity, disrupted neurotransmission, and BBB disruption, as well as the neurotoxic effects of drug abuse. All these factors by themselves or combinatorically contribute to neuroinflammation and neuronal damage, independent of the direct presence of viruses or viral proteins in the brain [5]. Neurons are known to be refractory to the virus and neuronal injury is known to occur primarily due to the presence of inflammatory cytokines released by virus-infected, activated microglial cells, in addition to the toxic viral proteins such as HIV Tat, Negative regulatory factor (Nef), and envelope glycoprotein gp120, as well as chemokines [5,9]. Interestingly, it has also been shown that cART alone can contribute to the neuroinflammatory milieu in the brain [14].

Neuroinflammatory processes are regulated by a network of non-coding (nc) RNAs that either upregulate or downregulate gene expression and affect mRNA translation. Their mechanism of action and regulatory pathways are not entirely understood; however, their mode of action involves interaction with other intracellular molecules, including DNA, RNA, and proteins, resulting in complexes that regulate various aspects of cell physiology [15]. Epigenetic modifications of the ncRNAs that occur throughout an individual’s lifespan have been implicated in playing a role in several age-related complications, including cellular senescence [16]. The dysregulation of these ncRNAs also plays a role in the neuropathogenesis of HIV infection and NeuroHIV [17,18]. For example, lncRNA MALAT1 and HIV-1-enhanced lncRNA have been shown to activate HIV transcription [19,20]. Studies have also shown that the HIV Tat protein, an early neurotoxic protein present in the brains and CSF of PLWH [21,22], can also upregulate the expression of lncRNA U1, thereby disturbing the homeostatic functions of neurons, including calcium homeostasis, mitochondrial oxygen reduction, and ATP synthesis [18]. Additionally, other ncRNAs, such as micro (mi)RNAs and circular (circ)RNAs, have also been shown to be endowed with gene regulatory properties. Herein, we review the different classes of ncRNAs and their probable roles in HIV infection, as well as in the pathogenicity of HIV-associated neuroinflammation in the context of NeuroHIV. The review also explores potential diagnostic and therapeutic implications for modulating NeuroHIV pathways.

## 2. Non-Coding (nc)RNAs

**Figure 1:**
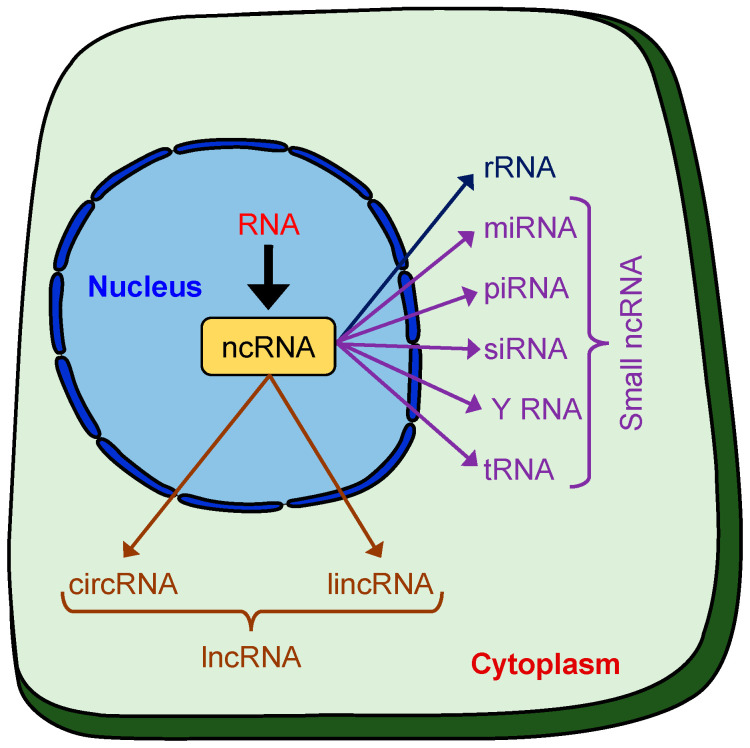
Schematic showing various types of non-coding RNAs, including miRNAs that regulate mRNAs, lncRNAs that modulate genes, siRNAs that guide RNA interference, piRNAs that silence transposons, circRNAs that act as sponges, Y RNAs involved in RNA stability, rRNAs that form ribosomes, and tRNAs that transport amino acids.

ncRNAs encompass a diverse group of transcripts that are transcribed from >70% of the human genome, but do not encode proteins. Increasing evidence in recent years suggests that ncRNAs play critical roles in a wide range of cellular processes in both health and disease [23,24,25,26,27,28]. More than 40 various types and sizes of ncRNAs have been identified over the years, including ribosomal RNAs (rRNAs), transfer RNAs (tRNAs), long non-coding RNAs (lncRNAs), microRNAs (miRNAs), circular RNAs (circRNAs), PIWI-interacting RNAs (piRNAs), and Y RNA (Figure 1). ncRNAs are further divided into housekeeping and regulatory types. Housekeeping linear ncRNAs, such as the tRNAs, rRNAs, small nuclear RNAs (snRNAs), and small nucleolar RNAs (snoRNAs), are reasonably well studied [29]. Regulatory ncRNAs, including miRNAs, small interfering RNAs (siRNAs), piRNAs, and circRNAs, on the other hand, have recently gained more attention for their roles in cellular regulation and gene transcription [30].

### 2.1. Mode of Action and Biological Functions

#### 2.1.1. microRNAs

More than one-third of human genes are regulated by miRNAs [31]. miRNAs are endogenous, short, single-stranded RNA molecules (18–25 nucleotides in length) that play critical roles in post-transcriptional gene regulation [32,33]. Most cognate miRNAs function by binding to complementary sequences in the 3′ untranslated region (UTR) of the target messenger RNA (mRNA) molecules, leading to translational repression or mRNA degradation. However, miRNA recognition elements can also be present in other parts of the mRNA transcript. Additionally, miRNA binding sites (miRNA response elements) have been detected in other mRNA regions, including the 5′-UTR, coding regions, or promoter regions [33,34]. Binding in these regions can either silence gene expression [35] or induce transcription [36], depending on the context. Recent studies have also suggested that almost 50% of mouse miRNAs (1079 out of 2049) contain at least one AU- or GU-rich 4-mer [25]. Notably, AU- or GU-rich, single-stranded RNAs have been shown to function as agonists for endosomal TLR7/8 RNA [37]. The AU- or GU-rich miRNAs bind to TLR7 and TLR8 in the endosomes and mediate the nuclear translocation of NFκB p65 from the cytoplasm [25,28], which, in turn, leads to cellular activation and toxicity.

**Figure 2:**
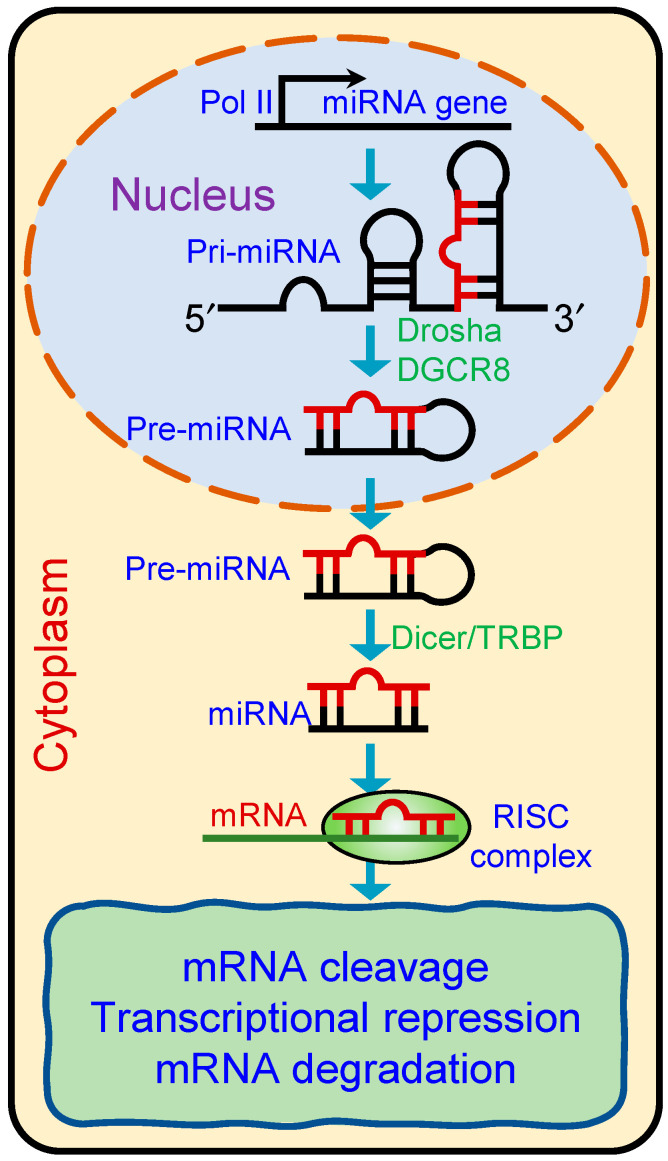
Schematic showing biogenesis of microRNAs. Primary miRNAs (pri-miRNAs) are transcribed in the nucleus and processed by the Drosha-DGCR8 complex into precursor miRNAs (pre-miRNAs). Pre-miRNAs are then exported to the cytoplasm, where Dicer/TRBP processes them into mature miRNA duplexes. One strand of the duplex is incorporated into the RNA-induced silencing complex (RISC), which guides the complex to target mRNAs for degradation or translational repression.

miRNAs are predominantly produced by canonical pathways from genomic DNA into long primary miRNA transcripts (pri-miRNAs) by RNA polymerase II or III [38]. This process is initiated by the microprocessor complex, comprising the RNA-binding protein DiGeorge Syndrome Critical Region 8 (DGCR8) and an endonuclease III enzyme, Drosha, in the nucleus. Drosha processes the pri-miRNA into precursor miRNAs (pre-miRNAs, 60–70 nucleotides), which are subsequently transported to the cytoplasm by a nuclear transport receptor, Exportin-5 [38]. In the cytoplasm, another endonuclease III enzyme, Dicer, along with its cofactor TRBP (HIV-1 TAR RNA-binding protein), cleaves the pre-miRNA to form miRNA duplexes consisting of mature miRNA and a complementary passenger strand (Figure 2). Both the strands are then loaded into the Argonaute protein and guided to the UTR region of target transcripts for gene regulation [39]. Non-canonical miRNA synthesis does not require Drosha- or Dicer-mediated cleavage. By regulating the expression of target genes, miRNAs play critical roles in various biological processes, including development, differentiation, cell cycle regulation, apoptosis, and immune responses.

#### 2.1.2. LncRNAs

LncRNAs constitute a diverse category of RNA molecules exceeding 200 nucleotides in length and may contain short open reading frames. They are typically transcribed by RNA polymerase II, resembling mRNAs in structure, with features like cap structures and poly(A) tails. Although less abundant than mRNAs, lncRNAs play crucial roles in diverse regulatory processes such as epigenetic modification, transcriptional and post-transcriptional gene regulation, chromatin dynamics, and nuclear organization [40,41].

Recent studies have illuminated the distinct characteristics of lncRNAs concerning their transcription, processing, export, and turnover. Despite their similarities with mRNAs in Pol II transcription, 5′-end m7G caps, and 3′-end poly(A) tails, lncRNAs exhibit unique properties influencing their cellular fates and functions. Their transcription often originates from genomic loci distinct from protein-coding genes. Phosphorylation-dysregulated RNA polymerase II leads to temporal chromatin accumulation and rapid degradation [42]. Moreover, lncRNAs undergo less efficient splicing than mRNAs due to weaker internal splicing signals and longer distances between splice sites, leading to enhanced nuclear retention [43,44]. Various factors influence the nuclear localization of lncRNAs, including their mode of transcription, internal splicing signals, and expression of splicing regulators. Some lncRNAs contain embedded motifs promoting nuclear retention, such as nuclear retention elements and repeat elements. While a significant fraction of lncRNAs is exported to the cytosol via the nuclear RNA export factor 1 pathway, others remain in the nucleus or localize to specific organelles like mitochondria and exosomes [45,46]. The turnover of lncRNAs is regulated by various molecular mechanisms, including RNA stability determinants and degradation pathways, which impact the functional dynamics of lncRNAs within the cells.

#### 2.1.3. CircRNAs

CircRNAs are a type of RNA molecule characterized by a covalently closed loop structure, lacking both 5′ caps and 3′ polyadenylated tails [47]. They are generated through a non-canonical splicing process called back-splicing, where a downstream splice donor site is joined to an upstream splice acceptor site, forming a circRNA [47]. CircRNAs are abundant in eukaryotic cells and have been identified across diverse species [48,49]. Once considered byproducts of splicing errors, circRNAs are now recognized as functional RNAs involved in various biological processes [50]. They can act as miRNA sponges, binding to and sequestering miRNAs to regulate gene expression. Additionally, circRNAs can interact with RNA-binding proteins, influence alternative splicing, and modulate transcription and translation [50]. The mechanisms of action of circRNAs are diverse and continue to be elucidated. Some of the well-described mechanisms by which circRNAs exert their functions include microRNA sponging [51,52], interactions with RNA-binding proteins (RBPs) and other regulatory proteins [50], regulation of transcription and RNA processing [53], modulation of translation [54], and scaffolding [55,56]. These mechanisms, in turn, endow circRNAs with diverse functions underlying gene regulation, cellular signaling, and disease pathogenesis via their interactions with RNA, proteins, and other biomolecules.

#### 2.1.4. Y RNAs

Y RNAs are a group of small non-coding RNAs initially identified as components of the Ro RNP complex [57]. Highly conserved across species, Y RNAs are typically 83 to 112 nucleotides in length [57]. They are transcribed by RNA polymerase III and are characterized by a conserved stem-loop secondary structure. Initially thought to be primarily involved in RNA stability and quality control, Y RNAs have been implicated in diverse cellular functions, including DNA replication, RNA splicing, and response to cellular stress [57,58]. Additionally, they have been shown to play roles in regulating cell proliferation and differentiation, suggesting their significance in developmental processes [59]. Despite their relatively small size, Y RNAs exhibit functional versatility and are emerging as important players in various aspects of cellular biology. A recent study by Marben et al. (2021) showed that Y RNA fragments (YF1) are secreted in the EVs released by cardiosphere-derived cells (CDCs). The mechanism of the cardioprotective role of EV-YF1 involved the induction of an anti-inflammatory cytokine interleukin 10 (IL-10) by EV-YF1 in macrophages [24]. Ongoing research in the field will enable the elucidation of the roles of Y RNAs and their regulatory mechanisms in both normal physiological processes and disease states.

## 3. Non-Coding RNAs in the Neuropathogenesis of HIV Infection and Related Comorbid Conditions

It is well recognized that there is a presence of latent HIV as a viral reservoir in the brains of PLWH, which is reactivated in the face of cART interruption [60]. HIV infection also causes a decline in CD4+ T cells, dampening the host’s immune responses and increasing the susceptibility of the host to opportunistic infections and tumorigenesis [61]. There is ample evidence in the field that CD4+ T cells, vital for combating viral infections, are intricately regulated by a complex network of ncRNAs [17,62]. ncRNAs play pivotal roles in various aspects of HIV pathogenesis, influencing viral replication, latency, regulation of immune response, and disease progression. Herein, we will focus on the role of two classes of ncRNAs—miRNAs and lncRNAs—in regulating the response to HIV infection, T cell activity, apoptosis, viral latency, and reactivation. We also highlight the role of various ncRNAs in HIV-associated comorbidities such as HIV-associated cancers and substance misuse.

### 3.1. Role of lncRNAs in HIV Infection and Neuropathogenesis

**Figure 3:**
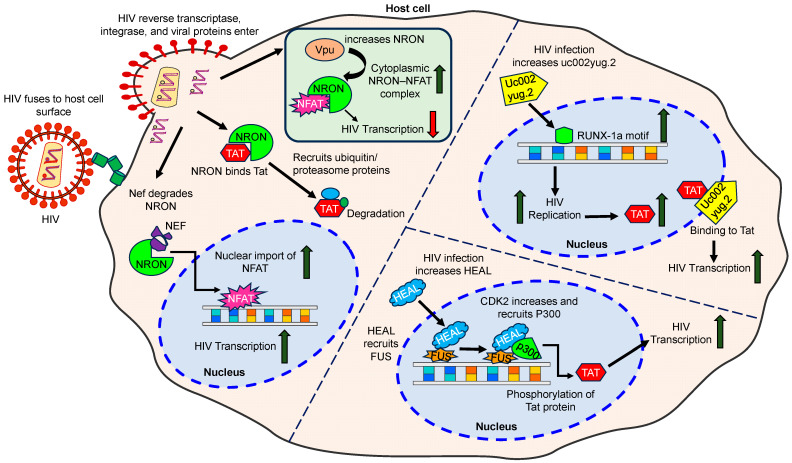
Schematic showing the interactions of HIV and HIV proteins, such as Tat, Nef, and Vpu, with lncRNAs such as NRON, uc002yug.2, and HEAL. These lncRNAs are involved in the regulation of HIV transcription, protein degradation, and HIV replication. These interactions illustrate the complex regulatory network between HIV/HIV proteins and host lncRNAs, as explained in detail in the text.

Several lncRNAs have been found to be dysregulated during HIV infection and they are believed to play a role in the pathogenicity of viral infection (Figure 3).

These lncRNAs include HEAL, GAS5, MALAT1, uc002yug.2, NEAT1, NKILA, LOC102549805, BACE1-AS, LINC00313, NRON, and LINC00173, which have been identified to influence the response of the immune system to HIV infection and its associated pathological effects (Table 1) [18]. Numerous studies have focused on the roles of lncRNAs in the pathogenesis of HIV infection involving different cell types in the CNS, such as macrophages, monocytes, microglia, astrocytes, and neurons. Additionally, lncRNAs can also impact the cellular response to HIV. LncRNAs such as GAS5, NEAT1, NRON, and 7SK are implicated in the repression of HIV transcription.

#### 3.1.1. LncRNA HEAL

A prominent study focused on the HIV-1-enhanced lncRNA (HEAL) expression profile in HIV-infected primary monocyte-derived macrophages and identified an increased expression of lncRNA HEAL in macrophages, T cells, and microglia [63]. This lncRNA was also found to increase in peripheral blood mononuclear cells isolated from blood samples of PLWH. Interestingly, HEAL serves as a broad enhancer for many strains of HIV-1, operating by binding with RNA-binding protein Fused in Sarcoma (FUS), thereby boosting HIV replication by recruiting histone acetyltransferase p300 to the HIV promoter. Additionally, the HEAL–FUS complex can elevate CDK2 expression. Recent research indicates that disrupting HEAL or inhibiting the formation of the HEAL–FUS complex can suppress HIV replication via epigenetic processes. Consequently, this approach has been proposed to eradicate viral reservoirs [63].

#### 3.1.2. LncRNA MALAT1

Metastasis-associated lung adenocarcinoma transcript 1 (MALAT1), originally discovered in metastatic carcinoma cells, has been implicated in HIV transcription. A recent study found that MALAT1 is overexpressed in HIV-infected CD4+ T-lymphocytes and enhances HIV transcription by preventing the binding of the core component Enhancer of Zeste Homolog 2 (EZH2) to the HIV long terminal repeat (LTR) promoter and by inhibiting the Polycomb Repressive Complex 2 (PRC2)-mediated methylation of histone H3 at lysine 27 (H3K27me3) [19]. The depletion of MALAT1 by CRISPR/Cas9 resulted in a significant reduction in gene transcription and viral replication driven by the HIV LTR. This suggests that targeting MALAT1, which promotes HIV transcription, could be a novel approach to treating HIV infection [19].

#### 3.1.3. LncRNA SAF

Apart from CD4+ T-lymphocytes, tissue-resident macrophages are also susceptible to HIV infection. This susceptibility allows them to harbor and facilitate HIV replication, thus making them resistant to virus-induced cell death. One study showed that lncRNA SAF regulated the apoptotic effector caspases in macrophages [65]. There was a significant increase in SAF expression in HIV-infected human monocyte-derived macrophages (MDMs) compared to the bystander or unexposed cells. Furthermore, reducing SAF expression using siRNA led to enhanced caspase-3/7 activity in HIV-infected MDMs, ultimately resulting in selective cell death in HIV-infected macrophages. These findings imply that lncRNA SAF could be a potential therapeutic target for combating the HIV reservoir in cells [65].

#### 3.1.4. LncRNA uc002yug.2

Among the limited number of lncRNAs known to be involved in HIV replication and latency is uc002yug.2. Originally identified as being highly expressed in esophageal squamous cell carcinoma, uc002yug.2 was found to enhance HIV replication, LTR activity, and the activation of latent HIV in cell lines and CD4+ T cells from HIV-infected individuals. A study by Huan et al. demonstrated that overexpressing lncRNA uc002yug.2 increased Tat protein expression, while downregulating lncuc002yug.2 RNA using the shRNA led to elevated levels of RUNX1b and RUNX1c. They also found that lncRNA uc002yug.2 contributed to HIV replication and latent HIV reactivation by reducing RUNX1b and RUNX1c expression, but not RUNX1a, while also increasing Tat expression. These findings underscore the potential of lncRNA as a therapeutic target, owing to its role in reactivating latent HIV [66].

#### 3.1.5. LncRNA GAS5

It has been shown that a decrease in lncRNA GAS5 is associated with the activation of DNA damage response [73]. This alteration influences the activity and apoptosis of CD4+ T cells isolated from PLWH by enhancing the expression of miRNA-21. This phenomenon has been observed in PLWH undergoing cART, who often exhibit immune activation due to low-grade inflammation. Mechanistically, lncRNA GAS5 reduces the expression of miRNA-21, a microRNA that regulates crucial signaling pathways involved in the DNA damage response. The persistent activation of T cells resulted in the downregulation of lncRNA GAS5, the upregulation of miRNA-21, the dysfunction of CD4+ T cells, and the induction of apoptosis in these cells. It has also been proposed that inhibiting the lncRNA GAS5:miRNA-21 axis could improve the function and survival of CD4+ T cells in PLWH receiving cART [73].

#### 3.1.6. LncRNA NEAT1

Several studies have independently investigated the expression and potential role of Nuclear paraspeckle assembly transcript 1 (NEAT1) in HIV-infected cells to elucidate its impact. In one study, the anti-HIV activity of lncRNA NEAT1 was demonstrated by using CRISPR/Cas9 to generate NEAT1 gene knockout Jurkat CD4+ T cell lines, which exhibited increased HIV replication compared to parental Jurkat cells [74]. These findings thus imply that HIV could exploit the downregulated expression of lncRNA NEAT1 in activated CD4+ T cells to enhance viral replication and facilitate infection. Another study demonstrated that lncRNA NEAT1 expression was altered following HIV infection. Knocking down NEAT1 using siRNA and antisense DNA increased viral production by promoting the export of Rev-dependent instability element-containing HIV mRNAs from the nucleus to the cytoplasm. These results highlighted the importance of NEAT1 in maintaining the structural integrity of the nuclear paraspeckle, a crucial subcellular organelle for HIV replication [68].

#### 3.1.7. LncRNA NRON

In contrast to the lncRNAs mentioned above, the non-coding repressor of the nuclear factor of activated T cells (NRON) has been shown to provide a mechanism for the differential regulation of HIV transcription at various stages of the HIV viral lifecycle. One prominent study showed that the early viral accessory protein Nef suppressed lncRNA NRON expression in human T cell line models Jurkat and J1.1, whereas the late protein Vpu had the opposing effect [69]. LncRNA NRON can degrade the Tat protein by linking it to ubiquitin/proteasome components, thereby restricting viral replication and transcription and promoting latency [75]. Conversely, the depletion of lncRNA NRON, especially when combined with a histone deacetylase inhibitor, resulted in the reactivation of viral production from latently infected primary CD4+ T-lymphocytes. These findings suggest that targeting lncRNA NRON regulation could be a promising strategy for developing agents that reverse latency in HIV [75].

#### 3.1.8. LncRNA BACE1-AS

LncRNA BACE1-AS positively influences the expression of β-secretase (BACE1) enzymes; their levels are regulated in normal physiological processes such as brain and vascular maintenance. A dysregulation of BACE1-AS is reported in several human diseases, including Alzheimer’s disease (AD), Parkinson’s disease (PD), heart failure, and mild cognitive impairments [76]. BACE1-AS has also been identified as a potential biomarker for distinguishing patients with AD from healthy individuals. In one study by Fotuhi et al., the levels of BACE1-AS mRNA were compared in the plasma versus plasma-derived exosomes from AD patients and healthy controls. While there was no clear link between BACE1-AS expression and the diseased state, patients with AD exhibited a higher BACE1-AS expression compared to those at the pre-AD stage, thus corroborating the likely potential role of BACE1-AS as an AD biomarker [77].

Intriguingly, the HIV Tat protein has been reported to upregulate the expression of host lncRNA BACE1-AS and the associated genes BACE1, APP, and Aβ isoforms in human primary astrocytes [70]. The underlying mechanism involved HIV Tat-mediated activation and translocation of the transcription factor, hypoxia-inducible factor (HIF-1α), to the nucleus, followed by its physical association with lncBACE1-AS. This complex binds to the BACE1 promoter, in turn, resulting in the increased expression of both BACE 1 and amyloid Aβ 1-42. A similar upregulation of HIF-1α and lncRNA BACE1-AS was also observed in the brains of SIV-infected macaques, particularly in the frontal cortex and hippocampus [70]. Increased levels of lncRNA BACE1-AS were also found in these brain regions of HAND patients, thereby underscoring the role of the HIF-1α:lncRNA BACE1-AS axis as a potential therapeutic target for treating NeuroHIV. Interestingly, a similar upregulation of the HIF-1α:lncRNA BACE1-AS axis was also observed in the brains of rhesus macaques who had been administered with chronic morphine [78,79], linking these molecular changes to amyloidosis [78]. An extension of this study also reported the presence of neurotoxic amyloid cargoes in brain-derived EVs from morphine-administered macaques, thus highlighting the role of EVs as peripheral biomarkers for AD [78].

#### 3.1.9. Other lncRNAs

Animal studies have also demonstrated that HIV Tat, the neurotoxic protein produced by HIV-infected cells, can increase the expression of lncRNA LOC102549805 (lncRNA U1). Elevated LOC102549805 levels in neurons disrupt the bioenergetic functions by affecting calcium homeostasis, mitochondrial oxygen reduction, and ATP synthesis [80]. Consequently, activating lncRNA-U1 by HIV Tat leads to mitochondrial damage in neurons, ultimately disrupting both the autophagy and apoptotic pathways [80]. The HIV Tat protein can also suppress the expression of LINC00313 and its interaction with PRC2, promoting cell invasion, migration, and angiogenesis [72]. The lncRNA NKILA (negative regulator of NFκB signaling) is also implicated in the response to HIV infection. The NFκB-associated induction of gene expression from the HIV-1 long terminal repeat (LTR) is crucial for HIV-1 transcription and reactivation from latency. LncRNA NKILA has been demonstrated to inhibit HIV-1 replication by modulating the NFκB pathway, thereby reducing the activity of the HIV-1 LTR promoter [81,82,83].

### 3.2. Role of miRNAs in HIV Infection and Neuropathogenesis

A dysregulated expression of miRNAs has been reported in various stages of HIV infection [18,84]. Cellular miRNAs can influence both HIV infectivity and replication. They can directly impact HIV infection by targeting viral RNA through base pair complementarity. Additionally, some miRNAs indirectly inhibit HIV-1 replication by targeting host proteins crucial for productive HIV-1 infection. More recently, a small number of miRNAs that enhance HIV-1 infection have been discovered [18,84].

#### 3.2.1. miRNA-155

miRNA-155, a known key regulator of T cell differentiation, has been implicated in modulating the course of HIV infection. The expression of miRNA-155 is elevated in CD8+ T cells from PLWH, particularly in discord controllers and those who are cART-naïve. The expression of this miRNA is positively associated with the proportions of effector and effector memory CD8+ T cells, while showing an inverse correlation with the proportions of naïve and central memory CD8+ T cells. Overall, the upregulation of miRNA-155 expression in CD8+ T cells of PLWH is associated with the differentiation of these cells [85]. The NFκB:miRNA-155 pathway can stimulate an immune response in monocyte-derived macrophages following the suppression of CCL2 [86].

#### 3.2.2. miRNA-146-a

The dysregulated expression of miRNAs can impact the response of the immune cells to HIV infection. Increased levels of miRNA-146a have been associated with a decreased expression of antiviral cytokines, cellular cytotoxic activity, and immune cell exhaustion [87]. Another study found that HIV infection resulted in the elevated expression of miRNA-146a, leading to a reduction in the release of CCL5 from both macrophages and monocytes [88].

#### 3.2.3. miRNA-191-5p

A comprehensive analysis of miRNA profiles in peripheral blood mononuclear cells (PBMCs) from HIV patients who were untreated, those receiving cART, and healthy individuals revealed significant differences in the levels of miRNA-191-5p among these groups. Importantly, miRNA-191-5p has been shown to suppress HIV replication in cellular model systems. CCR1 and NUP50 have been identified as targets of miRNA-191-5p, while silencing NUP50 has been shown to inhibit HIV-1 replication [89]. Overall, the expression of miRNA-191-5p is downregulated in the PBMCs of HIV-infected patients and this miRNA acts as a suppressor of HIV infection by regulating the expression of nucleoporin 50 [89].

#### 3.2.4. miRNA-103 and miRNA-107

Both miRNA-103 and miRNA-107 have been identified as regulators of the HIV co-receptor CCR5 expression, with their levels being increased in uninfected bystander HIV-exposed MDMs. The upregulation of miRNA-103 in MDMs reduced the expression levels of CCR5 and inhibited CCR5-dependent HIV entry. Furthermore, IL-1β stimulation has also been shown to increase the miRNA-103 expression in MDMs [90]. Of note, this cytokine also reduces CCR5 transcripts in IL-1β-treated MDMs. The upregulation of miRNA-103/107 levels is part of the p53 response induced by IL-1β, which confers the resistance of macrophages to HIV entry [90].

The potential of miRNAs to target and suppress key host dependency factors offers significant promise in inhibiting HIV replication. The activation or differentiation status of CD4+ T cells and monocytes/macrophages, the primary cell types infected by HIV-1 in vivo, determines their susceptibility to viral replication. Because miRNAs are expressed differentially in resting versus activated CD4+ T cells and monocytes versus macrophages, it is reasonable to envision that the physiological state of these cells plays a crucial role in determining how miRNAs affect viral replication. Below is a list of miRNAs that have been shown to target critical host dependency factors (HDFs) (Table 2).

#### 3.2.5. miRNAs in NeuroHIV

Several studies have indicated that during HIV infection, the expression of numerous miRNAs, including miRNA-7, miRNA-9, miRNA-34, and miRNA-138, are upregulated in astrocytes. In HIV Tat-exposed astrocytes and in the HIV transgenic rat model, miRNA-34a and miRNA-138 were shown to modulate astrocytic function by downregulating SIRT1 expression and promoting cellular senescence, thus implicating the role of HIV Tat protein in cellular senescence [101].

**Figure 4:**
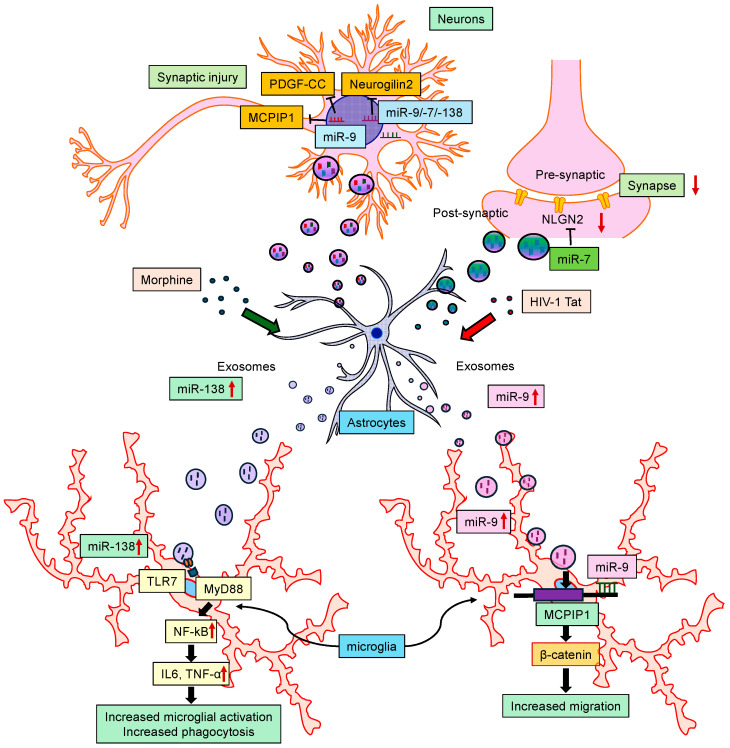
Schematic showing the interactions of miRNAs with central nervous system cells. Certain miRNAs modulate astrocytic function and induce cellular senescence. Also, some miRNAs released in extracellular vesicles (EVs) from astrocytes target neurons and microglia, contributing to synaptic alterations, microglial activation, and disease pathogenesis.

On the other hand, other miRNAs, such as miRNA-7, miRNA-9, miRNA-21, and miRNA-138, were released in EVs isolated from astrocytes and microglia [28,70,71,102,103]. Another study demonstrated that astrocytes, when exposed to HIV Tat protein, released EVs containing miRNA-7, which, in turn, was taken up and internalized by the neurons, ultimately resulting in the downregulation of its target Neuroligin 2 (NLGN2) and thereby contributing to the synaptic alterations [104]. In an independent study, it was also shown that miRNA-9 inhibited the expression of the anti-inflammatory Monocyte Chemotactic Protein-Induced Protein 1 (MCPIP1) [102] in both mouse primary microglia and in C57BL/6N mice, thus resulting in microglial activation. On the other hand, morphine-stimulated human A172 astrocytes released EVs enriched for miRNA-138 and were taken up by microglia, resulting in their activation. The mechanism of the EV-miRNA-138-mediated activation of microglia involved the binding of the GUUGUGU motif of miRNA-138 to the endosomal TLR7 and ensuring the activation of the NFκB pathway [70]. Additionally, the HIV Tat-mediated release of miRNA-9-enriched EVs from human A172 astrocytes was shown to induce a migratory phenotype in the microglia [103]. The exposure of microglia to astrocyte-EV-miRNA-9 led to the downregulation of the miRNA-9 target phosphatase and tensin homolog (PTEN) in microglia via binding to the 3′UTR seed sequence of the PTEN mRNA, eventually leading to microglial migration. Along these lines, EV-miRNA-21 released from macrophages was shown to be taken up by neurons, targeting neuronal TLR7 and promoting apoptosis [28]. Thus, these findings underscore the idea that communication between glial cells and neurons via miRNAs released through EVs contributes to the dissemination of disease pathogenesis (Figure 4).

### 3.3. ncRNAs in HIV-Related Comorbid Conditions

#### 3.3.1. HIV-Associated Cancers

HIV infection is recognized as a risk factor for several types of cancers, such as non-Hodgkin lymphoma, Kaposi’s sarcoma, and cervical cancer [105,106,107]. HIV itself can have direct oncogenic effects through the HIV Tat protein. These effects involve various mechanisms, including synergy with other oncogenic viruses, interference with the function of tumor suppressor genes, and disruption of cell cycle regulation [108,109,110]. Furthermore, the development of HIV-associated cancers is partially attributed to the dysregulation of lncRNAs that control the expression of viral and host genes. The involvement of lncRNAs in HIV-induced carcinogenesis may vary depending on the virus, as regulatory lncRNAs can originate from both the virus and the host cells. During infection, HIV alters the expression profiles of both host and viral lncRNAs [111]. In turn, HIV viral proteins are reported to cause carcinogenesis by regulating the lncRNA expression, as summarized in Table 3 below.

The expression of miRNA-144 has been found to be increased in alveolar macrophages isolated from HIV transgenic rats and in HIV-infected human MDMs, compared to their respective controls. It is suggested that inhibiting miRNA-144 or interfering with its impact on the antioxidant transcription factor Nrf2 could alleviate the HIV-associated dysfunction of alveolar macrophages and improve lung function in HIV-infected individuals [115].

#### 3.3.2. HIV Infection and Substance Abuse

Since the emergence of the HIV epidemic in the 1980s, HIV infection and substance use have been intricately interlinked, so much so that they have been referred to as intertwined epidemics. It is well recognized that the HIV disease severity and progression are potentiated by substance use, in turn significantly impacting HIV transmission, diagnosis, and treatment outcomes [116,117]. Clinical studies provide evidence that substance use comorbidity enhances viral load, hastens disease progression, and exacerbates AIDS-related mortality, even in individuals adhering to cART regimens [118]. Misused drugs can facilitate the entry of HIV into the brain, leading to dysfunctional immune response and the release of neurotoxins, ultimately leading to a state of chronic neuroinflammation [119].

Clinical studies have also demonstrated that PLWH who misuse cocaine exhibit worse behavioral and neurocognitive outcomes [120]. Furthermore, both HIV infection and substance misuse are known to dysregulate the expression of miRNAs and lncRNAs regulating gene expression [121,122,123]. In one study, the authors explored the expression profile of miRNAs and lncRNAs in the context of exposure to HIV Tat protein and cocaine to human primary astrocytes, using small and whole-RNA sequencing analyses. Herein, the authors demonstrated that two miRNAs (miRNA-2355 and miRNA-4726-5p), four lncRNAs (LINC01133, HHIP-AS1, H19, and NOP14-AS1), and two genes (NDUFA9 and LIPG) were significantly dysregulated in human primary astrocytes exposed to both HIV Tat and cocaine. This study demonstrated the role of the LINC01133:miRNA 4726-5p:NDUFA9 axis interactions, implicating it in HIV Tat and cocaine-mediated astrocytic dysfunction and associated neurodegeneration [120].

Findings from our lab have demonstrated elevated levels of miRNA-29b in the basal ganglia of SIV-infected rhesus macaques that were morphine-dependent, compared with SIV-infected control macaques. Additionally, this upregulation of miRNA-29b was likely mediated by an indirect effect on neurons, wherein the exosomes released from HIV Tat and morphine-stimulated astrocytes were enriched in miRNA-29b, which was taken up in the neurons (rat primary neurons and SH-SY5Y cells), ultimately resulting in neuronal death [124]. Furthermore, conditioned media from astrocytes (rat primary astrocyte and human astrocytoma A172 cells) stimulated with both HIV Tat and morphine reduced the expression of the neuronal growth factor—platelet-derived growth factor-B (PDGF-B)—resulting in increased neuronal apoptosis. This study thus implicated the role of miRNA-29b in HIV Tat and morphine-mediated neurodegeneration in NeuroHIV. The interaction between miRNA-29b and PDGF-B was confirmed by observing the binding of miRNA-29b to the 3′-UTR of PDGF-B mRNA, leading to the suppression of PDGF-B translation in SH-SY5Y cells [124].

In another study, we also showed the combinatorial effects of HIV Tat protein and cocaine on the DNA methylation of the miRNA-124 promoter and its potential role in microglial activation and neuroinflammation [125]. The downregulated expression of microglial miRNA-124, induced by HIV Tat and cocaine, contributed to increased microglial activation through the promoter DNA hypermethylation of miRNA-124, thus causing an increased release of proinflammatory cytokines such as IL-1β, IL-6, and tumor necrosis factor (TNF) [125]. Neuroinflammation is a critical factor underlying the development of HAND in PLWH. Activated microglia leading to increased release of proinflammatory cytokines are linked to cognitive impairment, memory and learning difficulties, and sensory impairments in HAND patients with a history of drug abuse [126,127,128,129]. It is thus plausible to speculate that miRNA-124 could be a possible target for dampening microglial activation in the context of HIV and cocaine exposure.

## 4. Diagnostic and Therapeutic Implications of ncRNAs in NeuroHIV

While there is an extensive scientific literature on understanding the role of miRNAs in human cancers, current studies are focused on identifying the roles of ncRNAs, such as lncRNAs and circRNAs, as contributors to disease pathogenesis. These ncRNAs are not only considered potential therapeutic targets but are also gaining momentum as valuable biomarkers. Pathogen exposure has been shown to regulate ncRNAs, which are crucial in modulating host genome expression. As a result, these regulatory molecules have become important targets for modifying disease outcomes [121]. Synthetic nucleic acid molecules, like siRNAs and antisense oligonucleotides (ASOs), are designed to target specific viral RNAs or host factors essential for HIV replication. siRNAs targeting the viral genes or host factors required for viral entry, integration, or transcription have been investigated as potential therapeutics [130]. ASOs targeting host factors involved in viral replication, such as host cell surface receptors or transcription factors, are also being explored [131,132,133]. ncRNAs are being investigated as therapeutic targets to modulate host immune responses against HIV. Certain miRNAs or lncRNAs regulate immune cell activation, cytokine production, or antigen presentation; targeting these ncRNAs can potentially enhance antiviral immune responses. CRISPR-Cas systems are also being developed to target the specific ncRNAs involved in HIV replication or host–virus interactions. New therapeutic approaches using CRISPR interference (CRISPRi) to silence viral or host ncRNAs implicated in HIV pathogenesis can also be considered as candidates in the future. Further research is needed to better understand the roles of different ncRNAs in HIV replication and neuropathogenesis and to develop safe and effective therapeutic approaches. Additionally, challenges such as delivery methods, specificity, and off-target effects need to be addressed for successful clinical translation.

### 4.1. LncRNAs as Therapeutic Targets for HIV1 and Drugs of Abuse-Related Disorders

Due to their specific expression in particular tissues and cells, lncRNAs present an appealing opportunity for therapeutic interventions. Several lncRNAs have been identified as potential targets for therapeutic interventions against HIV infection. Ongoing research is exploring the potential of lncRNAs in HIV therapeutics; however, the field is still in its infancy and clinical applications targeting lncRNAs in HIV therapy are yet to be established. Several lncRNAs have been studied for their roles in HIV infection and could likely serve as potential targets for future therapeutic interventions. For example, the knockdown of lncRNA NEAT1 is known to inhibit HIV replication in macrophages, suggesting its potential role as a therapeutic target for controlling HIV infection in these cells [68]. LncRNA MALAT1 knockout has also been shown to downregulate HIV-1 infection and transcription [19]. The blockade of HIV-1-enhanced lncRNA effectively impedes HIV-1 viral reactivation in both MDMs and T cells, upon discontinuing azidothymidine [63]. The recruitment of HIV-Encoded Antisense lncRNA to the 5′LTR has instigated epigenetic modifications in histones linked with the viral promoter, ultimately leading to the suppression of HIV-1 gene expression [134]. Trans-Activation Response RNA-gag (TAR-gag) is capable of suppressing HIV-1 transcription and maintaining latency by sequestering Tat and facilitating its degradation within the context of an interaction network involving Tat, Vpr, and Vif viral proteins with human proteins. The TAR-gag mechanism engages with the proteins responsible for transcriptional suppression, forming a dynamic RNA-protein complex that intricately governs the gene expression of HIV-1 [135].

Another lncRNA, lncRNA BACE1-AS, has been implicated in regulating BACE1, an enzyme involved in producing Aβ peptides. We have previously reported that both HIV Tat and morphine can induce the upregulation of HIF-1α in astrocytes, in turn, leading to an increased expression of lncRNA BACE1-AS, which regulates the expression of BACE1, as well as ensuing Aβ peptide accumulation. It can thus be envisioned that blocking lncRNA BACE1-AS could be developed as a therapeutic intervention aimed at preventing or mitigating AD-like neuropathology in both HIV-infected individuals, as well as opioid addicts [70].

HIV and substance abuse often intersect, leading to poorer health outcomes and an increased risk of HIV progression. The involvement of opiates in HIV disease progression has been extensively studied. Morphine is reported to further complicate neurocognitive impairment in HIV patients by altering immune function and exacerbating inflammation, potentially accelerating the progression of HAND. Functional impairment of microglial phagocytosis is a major contributor to neurocognitive damage. Findings from our lab have reported that long intergenic ncRNA Cox2 (lincRNA Cox2) is a key regulator of microglial phagocytosis, potentially through its effects on inflammatory signaling pathways [136]. Additionally, we demonstrated that administering astrocyte-derived EVs containing lincRNA Cox2 siRNA through intranasal delivery successfully reinstated microglial phagocytic activity in mice treated with morphine. These studies have implications for advancing the development of ncRNA-loaded EV-based therapeutics tailored for addressing diverse neurocognitive and drug abuse-related disorders [25].

### 4.2. LncRNAs in the Diagnosis of HIV/AIDS

In addition, plasma levels of lincRNA chr12:57761837-57762303 and lincRNA:chr2:165509129-165519404 are reported as markers for HIV-1 infection, whereas LincRNA chr5:87580664-87583451, XLOC_001148, and lincRNA chr10:128586385-128592960 are reported as markers for HIV-2 infection [137]. The presence of NEAT1 in plasma has also been reported as a biomarker of HIV-1 infection [138].

### 4.3. miRNAs as Therapeutic Targets for HIV1 and Drug Abuse-Related Disorders

Certain cellular miRNAs can target viral RNA or host factors critical for HIV replication, influencing viral gene expression, assembly, and release. Targeting these miRNAs could potentially inhibit viral replication and spread. Also, modulating miRNA expression has the potential to enhance antiviral immune responses or mitigate the immune dysregulation associated with chronic HIV infection. Strategies for targeting miRNAs in HIV therapy include using synthetic miRNA mimics to strengthen the activity of antiviral miRNAs or employing miRNA inhibitors to suppress the activity of miRNAs that promote viral replication or latency. Delivering miRNA-based therapeutics to target cells in the context of HIV infection presents challenges, including achieving cell-specific targeting, overcoming cellular and intracellular barriers, and minimizing off-target effects. Various delivery platforms, such as lipid nanoparticles, viral vectors, or exosome-based delivery systems, are being explored to address these challenges. We have reported that morphine exposure can contribute to neuroinflammation by affecting the integrity of the BBB through the modulation of astrocyte-derived EVs containing miRNA-23a and the subsequent loss of pericyte coverage [139]. We have also reported that HIV-1 Tat-induced astrocytic extracellular vesicle miRNA-7 could contribute to synaptic impairment, potentially exacerbating the cognitive deficits observed in PLWH [104]. It is thus plausible that inhibitors against these miRNAs can act as potential adjunctive therapeutic options. In another study, we demonstrated that miRNA-29, when delivered to neurons via exosomes, regulated the expression of neuronal growth factor genes in the context of both the HIV Tat and morphine exposure of astrocytes. By targeting specific mRNA transcripts, miRNA-29 could modulate neuronal signaling pathways and cellular processes implicated in neuronal dysfunction. This study underscores the potential of targeting this mechanism to develop novel therapeutic strategies involving exosome-mediated microRNA transfer aimed at mitigating NeuroHIV-associated neurotoxicity and opioid-induced neurodegeneration [124].

### 4.4. miRNAs in the Diagnosis of HIV/AIDS

Several studies have assessed the expression levels of miRNAs in HIV-infected individuals, revealing variability between plasma and infected cells. Despite this variability, miRNA expression levels hold promise in delineating the progression of HIV infection. For instance, miRNA-146b-5p and miRNA-150 exhibit dysregulation across different phases of HIV infection, both in plasma and PBMCs. Notably, miRNA-150 and miRNA-146b-5p are reported to be upregulated in plasma, but downregulated in PBMCs of HIV-infected patients during the AIDS phase [140]. miRNA-150 has been identified as a promising candidate biomarker for monitoring disease progression and treatment efficacy in HIV/AIDS. Through comparative analysis of miRNA-150 levels in PBMCs and plasma across various cohorts, including healthy donors, asymptomatic patients, symptomatic patients, individuals on cART, and those with drug-resistant strains, the authors observed a significantly differential expression of this miRNA among the groups [140]. In another study, Huang et al. identified the upregulation of miRNA-28, miRNA-125b, miRNA-150, miRNA-223, and miRNA-382 as biomarkers of HIV-infected cells during the latency phase [141]. These miRNAs target the 3′ end of HIV mRNAs, thereby restraining virus production in infected resting CD4+ T cells. This review, thus, underscores the significance of miRNAs in diagnosing HIV-1 infection and assessing disease progression.

## 5. Conclusions and Future Perspectives

This review highlights the crucial roles of ncRNAs in regulating the molecular and cellular pathways linked with HIV infection, NeuroHIV, and associated comorbidities. Despite efforts to characterize ncRNAs for diagnosing and managing HIV infection and HIV-associated neuropathology, challenges remain in understanding their roles due to the complexity of these disorders involving multiple receptors, targets, and pathways [142,143,144]. Certain ncRNAs have specific expression patterns that are developmental or tissue-specific, thereby playing vital roles in maintaining tissue function and identity [145]. While several ncRNAs found in various biological components and body fluids have been implicated to show promise as diagnostic biomarkers for NeuroHIV, comprehensive assessments of ncRNA expression profiles are warranted for the diagnosis and management of HIV infection and NeuroHIV.

Although the mechanism(s) of small ncRNAs like miRNAs are well understood, the functions of lncRNAs are still being explored. LncRNAs significantly regulate cellular pathways and are essential in various differentiation and developmental processes [146,147,148]. Their roles in HIV-1 infection, NeuroHIV, and related comorbidities are rapidly emerging. Notably, miRNA-146a, miRNA-155, and miRNA-181a are implicated in microglia-mediated neuroinflammation, highlighting their importance in NeuroHIV and necessitating further research [149,150,151,152]. It can be envisioned that modulating ncRNAs could help revert glial cells to a neuroprotective state in neuroinflammation. Additionally, ncRNAs, or their antisense counterparts, packaged into exosomes (enabling them to cross the BBB [153]), can be considered promising therapeutic targets for neuroinflammation. NcRNA–exosome combinations could thus hold potential as gene therapy candidates, thus paving the way for precision, personalized NeuroHIV treatment.

Despite the promising potential of ncRNAs in treating HIV infection and its comorbidities, significant challenges remain. Issues include off-target effects, immunogenicity, poor cellular uptake, rapid degradation, and clearance [154,155]. Off-target effects are particularly problematic, causing side effects and reduced efficacy in miRNA-based therapies [156,157]. The CRISPR/Cas9 system offers a solution for ncRNA-related genome regulation or editing, minimizing off-target effects [158,159,160,161,162,163,164,165]. Exploring CRISPR/Cas9 in the NeuroHIV context is thus warranted. Additionally, determining dosage, safety, administration route, and treatment regimen for ncRNA delivery continues to be a challenge and needs to be overcome [151,166,167]. The development of techniques such as lipid-based nanoparticle encapsulation and cell-penetrating peptides could improve ncRNA stability and reduce immunological effects, as has been demonstrated for anticancer and COVID-19 therapeutics [168,169]. While these approaches are still nascent for HIV therapy, efforts should be aimed to develop them for future use.

In conclusion, ncRNAs hold promise for developing non-invasive biomarkers and treatments for HIV infection and its comorbidities. Advancements in ncRNA-based diagnosis, monitoring, and therapy could revolutionize disease management and significantly enhance the quality of life of the patients.

## Figures and Tables

**Table 1:** List of human lncRNAs in HIV infection and neuropathogenesis.

LncRNAs	Gene ID	Chromosomal Location	Target	Expression	Role in HIV Pathogenesis	Potential Applications
HEAL	111216282	1p35.3	FUS	Upregulation	Epigenetic regulation causes increased HIV transcription [63]	Inhibition of HEAL-FUS complex could suppress viral replication
MALAT1	378938	11q13.1	EZH2	Upregulation	Reduces histone methylations and enhances HIV transcription [64]	MALAT1 depletion by CRISPR/Cas9 reduces viral replication
SAF	100302740	10q23.31	Caspase 3/7	Upregulation	Reduces caspase 3/7 levels, increases viral replication [65]	Reducing SAF expression caused selective apoptotic cell death in infected cells
uc002yug.2	100506385	21q22.12	RUNX1	Upregulation	Increases Tat protein and HIV transcription [66]	Targeting uc002yug.2 by using shRNA reduces viral replication
GAS5	60674	1q25.1	miRNA-873	Downregulation	Inhibits HIV replication [67]	Inhibition of GAS5:miRNA-21 axis could improve the survival of CD4+ T cells
NEAT1	283131	11q13.1	PSF, p54^nrb^	Downregulation	Paraspeckle formation and retention of nuclear HIV transcripts [68]	Targeting NEAT1 to reduce HIV replication could be beneficial
NRON	641373	9q33.3; 9	NFAT	Downregulation by Nef and upregulation by Vpu	Inhibits Tat protein and decreases HIV transcription [69]	Targeting NRON expression could be used to reverse HIV latency
BACE1-AS	100379571	11q23.3	BACE1, APP, HIF1-α	Upregulation	Causes amyloidosis by Tat protein-induced upregulation of HIF1-α [70]	Targeting the levels of BACE1 could regulate HIV replication
NKILA	105416157	20q13.31	Gag p55	Downregulation	Reduces HIV-1 LTR promoter activity and viral replication [71]	Regulating NKILA levels could be used for inhibiting HIV replication
LINC00313	114038	21q22.3	PRC2	Downregulation	Inhibited by Tat protein and initiates cell invasion, migration, and angiogenesis [72]	Targeting LINC00313 could reduce HIV replication

**Table 2:** List of miRNAs targeting specific host dependency factors during HIV infection.

miRNAs	HDFs	Model System	Expression	Implications for HIV Infection	Potential Applications
miRNA-181	SAMHD1	THP-1, Jurkat cells	Downregulation	Increases the mRNA expression of SAMHD1, thereby increasing HIV replication [91]	Targeting miRNA-181 levels could reduce HIV replication
miRNA-132	p300	Jurkat cells	Upregulation	Upregulates post-CD4^+^ T cell activation and binds to p300 to suppress the host’s innate immune response, thus increasing HIV replication [92]	Inhibition of miRNA-132 could have therapeutic potential
miRNA-34a, miRNA-124a, Let7-c	p21, TASK1	HeLa-CCR5 cells	Upregulation	Post-infection, these miRNAs become upregulated, leading to the downregulation of p21/TASK1. This process enhances virion release and increases HIV replication [93]	Inhibition of these miRs could be detrimental on viral replication
miRNA-155	TRIM32	J-Lat 5A8 cells	Upregulation	Inhibits the HIV-activating effects of TRIM3 and NFκB, thereby promoting HIV latency [94]	Targeting miR-155 to reverse viral latency
miRNA-20a, miRNA-17-5p	PCAF	HeLa, HEK293, Jurkat, U1, PBMCs	Downregulation	Modulates pCAF and inhibits HIV proviral transcription [95]	Targeting these miRs to inhibit viral transcription
miRNA-34a, miRNA-155	SIRT1	TZM-bl, MAGI cells	Upregulation	Downregulates SIRT1, thereby altering HIV Tat function [96,97]	Augmenting SIRT1 by downregulation of these miRs could have therapeutic potential
miRNA-27b, miRNA-29b, miRNA-150, miRNA-198, miRNA-223	Cyclin T1	MM6 cells	Downregulation	Inhibits cyclin T1, thereby inhibiting HIV replication [98,99]	Targeting these miRs to inhibit cyclin T1 could reduce viral replication
miRNA-1236	DCAF1	Monocyte-derived dendritic cells	Upregulation	Inhibits HIV Vpr function [100]	Targeting miRNA-1236 to inhibit viral replication

**Table 3:** Effects of viral protein on lncRNA in HIV-associated comorbid conditions.

HIV Protein	Mechanism of Action	LncRNA	Types of Cancer
Tat	Promotes carcinogenesis and angiogenesis	LINC00313	Kaposi’s sarcoma [66]
Tat	Regulation of HIV reactivation	uc002yug.2	Cervical cancer [72]
Nef	Increases HIV replication and breast cancer tumorigenesis	NRON	Breast cancer [69,112]
gp120	Dysregulation of lincRNA-p21 to effect apoptosis	lincRNA p21	Glioblastomas [113,114]

## Data Availability

Not applicable.
